# The *Firmicutes/Bacteroidetes *ratio of the human microbiota changes with age

**DOI:** 10.1186/1471-2180-9-123

**Published:** 2009-06-09

**Authors:** D Mariat, O Firmesse, F Levenez, VD Guimarăes, H Sokol, J Doré, G Corthier, J-P Furet

**Affiliations:** 1 INRA, U910, Unité d'Ecologie et du Système Digestif, Domaine de Vilvert, 78350 Jouy-en-Josas, France; 2 INRA, UR496, Unité d'Immuno-Allergie Alimentaire, Domaine de Vilvert, 78350 Jouy-en-Josas, France; 3 Service de Gastroentérologie et Nutrition, Hôpital Saint Antoine, AP-HP, Paris France

## Abstract

**Background:**

In humans, the intestinal microbiota plays an important role in the maintenance of host health by providing energy, nutrients, and immunological protection. Applying current molecular methods is necessary to surmount the limitations of classical culturing techniques in order to obtain an accurate description of the microbiota composition.

**Results:**

Here we report on the comparative assessment of human fecal microbiota from three age-groups: infants, adults and the elderly. We demonstrate that the human intestinal microbiota undergoes maturation from birth to adulthood and is further altered with ageing. The counts of major bacterial groups *Clostridium leptum, Clostridium coccoides*, *Bacteroidetes, Bifidobacterium, Lactobacillus *and *Escherichia coli *were assessed by quantitative PCR (qPCR). By comparing species diversity profiles, we observed age-related changes in the human fecal microbiota. The microbiota of infants was generally characterized by low levels of total bacteria. *C. leptum *and *C. coccoides *species were highly represented in the microbiota of infants, while elderly subjects exhibited high levels of *E. coli *and *Bacteroidetes*. We observed that the ratio of *Firmicutes *to *Bacteroidetes *evolves during different life stages. For infants, adults and elderly individuals we measured ratios of 0.4, 10.9 and 0.6, respectively.

**Conclusion:**

In this work we have confirmed that qPCR is a powerful technique in studying the diverse and complex fecal microbiota. Our work demonstrates that the fecal microbiota composition evolves throughout life, from early childhood to old age.

## Background

The composition of the intestinal microbiota plays a significant role in human immunology, nutrition and pathological processes [1]. Describing the complexity and ecology of the intestinal microbiota is important for defining its effects on overall human health. This level of understanding has been hindered by the limited sensitivity and inherent biases of culture-based techniques. Recently, the study of the gut microbiota has received renewed interest due to the development of molecular methods for more accurately assessing its composition and diversity, formerly thought to contain a mere 400–500 bacterial species [2]. Bacterial strains which are not cultivable under conventional methods have thus been identified [3]. This has markedly increased the information available concerning the complexity of the human bowel microbiota, from which over 1250 Operational Taxonomic Units have been identified corresponding to several dominant phyla [4,5].

Although the microbiota in adults has been extensively studied, investigation into structural changes and compositional evolution from infants to the elderly has only recently begun. Very little information is available pertaining to possible variations that occur with ageing. In healthy adults, 80% of the identified fecal microbiota can be classified into three dominant phyla: *Bacteroidetes, Firmicutes *and *Actinobacteria *[6]. In general terms the *Firmicutes *to *Bacteroidetes *ratio is regarded to be of significant relevance in human gut microbiota composition [7]. On a more refined level, however, the fecal microbiota is a highly complex and diverse bacterial ecosystem. Within this ecosystems exists a hierarchy of dominant (> 10^9 ^Colony Forming Units (CFU)/g)) anaerobic bacteria, represented by the genera *Bacteroides, Eubacterium*, *Bifidobacterium, Peptostreptococcus, Ruminococcus, Clostridium and Propionibacterium*, and sub-dominant (< 10^9 ^CFU/g), bacteria of the Enterobacteriaceae family, especially *E. coli*, and the genera *Streptococcus, Enterococcus, Lactobacillus, Fusobacterium, Desulfovibrio *and *Methanobrevibacter *[8].

Establishment of the intestinal microbiota has been shown to be a progressive process [9]. This process of increasing diversity is required for proper development and is important for overall health. The major functions attributed to the microbiota present in the gut begin to manifest at the end of the second year of life and comprise: i) nutrients absorption and food fermentation [10], ii) stimulation of the host immune system [11] and iii) barrier effects against pathogens [12]. Once climax composition is achieved near the end of adolescence, this ecosystem displays a high stability in healthy adults [13]. Although the intestinal microbiota is relatively stable throughout adult life, recent studies indicated that modifications occur in the composition in elderly individuals. For example, a reduction in the numbers of *Bifidobacteria *and *Bacteroides *has been observed, accompanied also by a decrease of *Lactobacilli*. A commensurate increase in the number of facultative anaerobes also highlights the variation between adults and elderly individuals [14-17]. Such variation was also observed by Ley *et al. *[7] when a correlation between body weight and gut microbial ecology was analysed. The microbiota in obese subjects shows an elevated proportion of *Firmicutes *and a reduced population of *Bacteroides. *Conversely, a decreased *Firmicutes*/*Bacteroidetes *ratio has been directly related to weight loss [7].

The work presented here aims to continue to expand our understanding of the intestinal flora including its establishment, composition, and evolution. To that end, we focused on the important ratio between *Firmicutes *and *Bacteroidetes*. We used a qPCR-based approach to enumerate changes in bacterial populations in the human intestine.

This work is part of the few culture-independent studies [18] which use adapted molecular approaches to analyze modulations of fecal microbiota related to ageing.

## Results

### Microbiota specificities related to age

Average bacterial counts for each human age-group are summarized in Table 1. In adults, the *Bacteroidetes *and *Firmicutes *are the most prevalent phyla present, the latter of which combines the values obtained for the dominant *C. leptum *and *C. coccoides *groups and the sub-dominant *Lactobacillus *group. The *Bifidobacterium *genus is present in eight to ten-fold lower numbers than the two major phyla. *E. coli *was found to be present at 7.7 log_10 _CFU/g, also consistent with its characteristic sub-dominant population in adults.

**Table 1:** Composition of the human microbiota compared in three age groups

		TaqMan detection					SYBR-Green detection	
			
			*Firmicutes*	*Firmicutes*				*Firmicutes*
							
	n	All-bacteria (a)	*C. leptum *group (b)	*C. coccoides *group (b)	*Bacteroides/Prevotella *group (b)	*Bifidobacterium *genus (b)	*E. coli *(b)	*Lactobacillus/Leuconostoc/Pediococcus *group (b)
Infant	21	10.7 ± 0.1 (A)	-3.2 ± 0.4 (A)	-3.2 ± 0.4 (A)	-1.5 ± 0.3 (A)	-0.6 ± 0.2 (A)	-1.5 ± 0.3 (A)	-3 ± 0.2 (A)

Adult	21	11.5 ± 0.1 (B)	-0.7 ± 0.1 (B)	-1.2 ± 0.1 (B)	-1.5 ± 0.1 (AB)	-2.3 ± 0.3 (B)	-3.8 ± 0.1 (B)	-3.9 ± 0.3 (AB)

Elder	20	11.4 ± 0.1 (B)	-1.1 ± 0.1 (C)	-1.8 ± 0.1 (A)	-1 ± 0.1 (A)	-2.3 ± 0.3 (B)	-2.4 ± 0.2 (C)	-4.2 ± 0.2 (B)

n represents the number of samples in each group.

(a) All-bacteria results obtained by qPCR were expressed as the mean of the log_10 _value ± SEM.

(b) Results were expressed as the mean of the log_10 _value ± SEM of normalized data calculated as the log of targeted bacteria minus the log of All-bacteria number.

The non parametric Wilcoxon test was performed.

Data not sharing the same letter within a column are significantly diferrent at p < 0.05.

Quantification of samples from infants showed total bacterial counts to be nearly ten-fold lower in log_10 _values (10.7) than in adults and seniors (11.5 and 11.4, respectively). It is worth noting that while they constitute the major dominant groups in adults and elderly, *C. leptum *and *C. coccoides *groups are only observed at a sub-dominant level in infants. *Bifidobacteria *was clearly the most abundant group measured in infants. Owing to lower overall numbers of bacteria in infants, the *Bifidobacterium *genus represented a major fraction of the dominant bacterial species found in the infant fecal microbiota, far above *Firmicutes *and *Bacteroidetes*. Infants were also found to harbor an *E. coli *population at a level characteristic of a dominant group, 10^9 ^CFU/g, contrary to the level observed in adults.

### Normalized quantitative PCR data

When normalized against all bacterial group counts, the qPCR data (Table 1) can be represented as a percentage of total bacterial counts. Statistical analysis of the data show that *C. leptum*, and *C. coccoides *levels are significantly lower in infants (-3.2 and -3.2 Δlog_10 _respectively) than in adults (-0.7 and -1.2 Δlog_10 _respectively), while *Bacteroides *levels are equivalent in each age group. Alternatively, *Bifidobacterium *levels are greater in infants (-0.6 Δlog_10_) than in adults (-2.3 Δlog_10_) and seniors (-2.3 Δlog_10_). *Lactobacillus *counts are greater in infants (-3 Δlog_10_) than in seniors (-4.2 Δlog_10_) with an equivalent value in adults (-3.9 Δlog_10_). Interestingly, *E. coli *levels exhibit a progression between the three age groups since the highest counts are found in infants (-1.5 Δlog_10_), then decrease in adults (-3.8 Δlog_10_), ultimately stabilizing at an intermediate level in seniors (-2.4 Δlog_10_).

Finally, analysis of each bacterial population revealed no significant differences for the elderly when compared with those for adults with the exception of *C. leptum*, *C. coccoides *and *E. coli*, which as in infants, showed counts characteristic of a dominant group.

### *Firmicutes/Bacteroidetes *ratio

**Figure 1:**
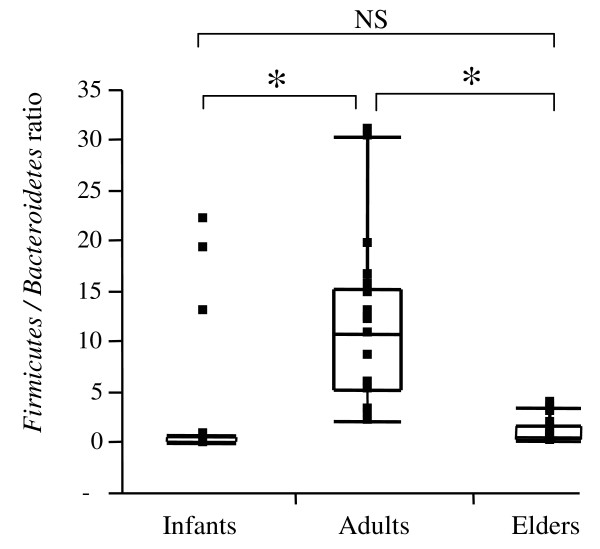
**Box-and-Whisker plot of *Firmicutes/Bacteroidetes *ratios in the three age-groups**. Horizontal lines represent the paired comparison. Boxes contain 50% of all values and whiskers represent the 25^th ^and 75^th ^percentiles. Significantly different (P < 0.05) ratios are indicated by *, while NS corresponds to non-significant differences.

For the *Firmicutes*/*Bacteroidetes *ratio, we observed significant differences between infants and adults (0.4 and 10.9, respectively) and between adults and elderly (10.9 and 0.6, respectively) (Figure 1). Notably, no significant differences were found between infants and elderly.

## Discussion

The microbiota of the large intestine plays an important role in host metabolism and maintenance of host health [19]. The accurate description of this bacterial community is an important question that has long remained a challenge owning to the limitations of culturing and isolation techniques. We have thus employed current molecular methods, i.e. quantitative PCR, to tackle this problem. Our work has allowed for a detailed description of the complex composition of the human intestinal microbiota which can serve as a basis to monitor gut microbiota changes in connection with diet or health.

Our results defining a standard adult profile, together with previous reports, showed that *C. leptum*, *C. coccoides, Bacteroides *and *Bifidobacterium *represent the four dominant groups of the adult fecal microbiota [8,20,21]. Sub-dominant groups are *Lactobacilli Enterobacteriaceae*, *Desulfovibrio*, *Sporomusa*, *Atopobium *as well as other bacterial groups including *Clostridium *clusters XI, XIVb, and XVIII [21,22].

Total bacterial counts overall were found to be significantly lower in infants than in adults and seniors. In infant fecal microbiota, we observed *Bifidobacterium *as the dominant group. This population dominance has been documented as a conserved feature during early gastrointestinal tract colonization [23]. Moreover, this observation is strongly related to diet, being enhanced by breast feeding [24,25]. In order to account for this observed effect, our infant panel consistent of 21 individuals aged 1 to 10 months, 14 of which were breast-fed, and 7 which were formula-fed. The older infants in our study received a more diverse diet. Significant higher numbers of *Bifidobacterium *were observed in infants versus adults and seniors. We conclude, therefore, that the high level of *Bifidobacterium *observed in our panel was not strictly correlated to breast feeding and could be considered as a broad signature of the infant microbiota during the first year of life.

This observation confirms previous reports indicating that the gastrointestinal tract is first colonized by facultative anaerobes, such as *E. coli *[23]. Strict anaerobes, such as *Clostridium*, colonize at later stages, as can be seen by the relatively low levels of *C. leptum *and *C. coccoides *in infants [23]. Our results are in agreement with these previous reports. We hypothesize that diet change must be considered among the primary causes for such a shift of microbiota between infants and adults.

In the case of elderly subjects, our qPCR results indicated a significant increase in the counts of *E. coli *when compared to adults. This data is consistent with other publications indicating that elderly subjects harbor a different *E. coli *microbiota profile compared to younger adults [26-28].

Concerning the microbiota of the elderly, a number of authors reported a reduction in the numbers and diversity of many protective commensal anaerobes, such as *Bacteroides *and *Bifidobacteria*. These reports also suggest a shift in the dominant bacterial species [17,19]. The *Firmicutes *to *Bacteroidetes *ratio was already shown to be of significant relevance in signaling human gut microbiota status [7]. This previous work focused on lean individuals and used 16S ribosomal RNA gene sequencing. Our measurements of the *Firmicutes*/*Bacteroidetes *ratio in adults obtained by our species-specific qPCR are in agreement with those obtained by Ley *et al. *[7]. Compared with young adults, the elderly have a different digestive physiology, characterized at a physiological level by a reduction in transit and of digestive secretions. These changes could explain the observed changes in the fecal microbiota associated with advancing age.

## Conclusion

Our results illustrate a measurable progression of bacterial species colonizing the human intestinal tract during different stages of life. This progression is easily observed and quantified using qPCR to evaluate numbers of bacteria belonging to major dominant and subdominant groups of the human fecal microbiota. The *Firmicutes/Bacteroidetes *ratio undergoes an increase from birth to adulthood and is further altered with advanced age. This ratio appears applicable in highlighting variations between infants, adults and the elderly. It can be linked to overall changes in bacterial profiles at different stages of life.

## Methods

### Sample collection

Fecal samples from 21 adults (25 to 45 years old) were recovered from previous sampling [20]. Fresh fecal samples were obtained from 21 infants (3 weeks to 10 months old) and 20 elderly subjects (70 to 90 years old). Infants in the study group were currently feeding with either breast milk (n = 16) or formula (n = 7). None of the infant subjects had been exposed to antibiotics. Adult and elderly subjects consumed an unrestricted Western-type diet. All subjects from these two age classes were not under antibiotic treatment or taking any other drugs known to influence the fecal microbiota composition for at least three months prior to sampling. All subjects were free of known metabolic or gastrointestinal diseases. Whole stools were collected in sterile boxes and immediately stored at 4°C under anaerobic conditions using an Anaerocult^® ^A (Merck, Nogent sur Marne, France). Samples were frozen within 4 hours at -20°C as 200 mg aliquots and stored for further analysis. Adults and elderly subjects were volunteers. Parents of infants gave written informed consent for this work. All procedures were approved by an ethics committee.

### DNA extraction

DNA was extracted from the 200 mg aliquots of feces as described previously [29,30]. After the final precipitation with isopropanol, nucleic acids were centrifuged and pellets were suspended in 225 μl of phosphate buffer and 25 μl of potassium acetate. After the RNase treatment, DNA was recovered by centrifugation and pellet was suspended in TE buffer.

### Real-time qPCR

Real-time qPCR was performed using an ABI 7000 Sequence Detection System apparatus with system software version 1.2.3 (Applied-Biosystems) [20,31]. Total numbers of bacteria were inferred from averaged standard curves as described by Lyons *et al*. [32].

TaqMan^® ^qPCR was adapted to quantify total bacteria populations in addition to the dominant (<1% of faecal bacteria population) bacterial species *C. coccoides*, *C. leptum*, *Bacteroides/Prevotella *and *Bifidobacterium*. qPCR using SYBR-Green^® ^was performed for the sub-dominant bacterial species *Escherichia coli *and for the *Lactobacillus/Leuconostoc/Pediococcus *group. Primers and probes used in this study were designed based on 16S rRNA sequences. A detailed description can be found in Furet *et al *[20] and Firmesse *et al *[31].

### Normalization of quantitative PCR data

Normalization was done by subtracting the value obtained for the "all bacteria" group from the values for the other bacterial groups in our study [20].

### *Firmicutes/Bacteroidetes *ratios

An estimation of the total amount of *Firmicutes *was obtained by adding bacterial values obtained from *C*. *coccoides, C. leptum *and *Lactobacillus*. For *Firmicutes/Bacteroidetes *ratios, calculations were obtained for each individual using CFU counts.

### Statistics

The non-parametric Wilcoxon test was performed using JMP^® ^software (Abacus Concepts, Berkeley, CA). The comparative results of *Firmicutes/Bacteroidetes *ratios were visualized as box-and-whisker plots showing: the median and the interquartile (midspread) range (boxes containing 50% of all values), the whiskers (representing the 25^th ^and 75^th ^percentiles) and the extreme data points. Statistical significance was accepted at *P *< 0.05.

## Authors' contributions

DM, FL and JPF carried out all PCR experiments. OF performed statistical studies. HS and VDG helped to draft the manuscript with the assistance of all authors. JD and GC conceived and coordinated the study. All authors read and approved the manuscript.
